# COVIDOA: a novel evolutionary optimization algorithm based on coronavirus disease replication lifecycle

**DOI:** 10.1007/s00521-022-07639-x

**Published:** 2022-08-26

**Authors:** Asmaa M. Khalid, Khalid M. Hosny, Seyedali Mirjalili

**Affiliations:** 1 Department of Information Technology, Faculty of Computers and Informatics, Zagazig University, Zagazig, 44519 Egypt; 2 Centre for Artificial Intelligence Research and Optimization, Torrens University Australia, Fortitude Valley, Brisbane, QLD 4006 Australia

**Keywords:** Coronavirus, Optimization, Frameshifting, Best cost, Convergence, Evolutionary algorithm

## Abstract

This paper presents a novel bio-inspired optimization algorithm called Coronavirus Optimization Algorithm (COVIDOA). COVIDOA is an evolutionary search strategy that mimics the mechanism of coronavirus when hijacking human cells. COVIDOA is inspired by the frameshifting technique used by the coronavirus for replication. The proposed algorithm is tested using 20 standard benchmark optimization functions with different parameter values. Besides, we utilized five IEEE Congress of Evolutionary Computation (CEC) benchmark test functions (CECC06, 2019 Competition) and five CEC 2011 real-world problems to prove the proposed algorithm's efficiency. The proposed algorithm is compared to eight of the most popular and recent metaheuristic algorithms from the state-of-the-art in terms of best cost, average cost (AVG), corresponding standard deviation (STD), and convergence speed. The results demonstrate that COVIDOA is superior to most existing metaheuristics.

## Introduction

Nature is full of principles and mechanisms that inspire scientists to develop complex computational problems [15]. Researchers developed various renature-inspired algorithms such as Genetic Algorithm (GA) [26] and Differential Evolution (DE) [63] over the years. These algorithms are based on the theory of natural evolution. Another group of Algorithms mimics the behavior of birds, animals, insects, plants, or fish, such as Particle Swarm Optimization (PSO) [44], Artificial Bee Colony (ABC) [41], Chicken Swarm Optimization (CSO) [48], Flower Pollination Algorithm (FPA) [75], Grey Wolf Optimization (GWO) [51], Whale Optimization Algorithm (WOA) [50], Cuckoo Search (CS) [76], Bird Mating Optimizer [6], Social Spider Optimization (SSO) [38], Krill Herd [25], and Seagull Optimization Algorithm (SOA) [19]. Other algorithms based on physical phenomena such as Water Cycle Algorithm (WCA) [22], Central Force Optimization (CFO) [24], Gravitational Search Algorithm (GSA) [60], Water Wave Optimization (WWO) [78], and Gradient-based Optimizer (GBO) (Ahmadianfar et al. 2020). Many other optimization algorithms are proposed by [5, 7, 10, 11, 13, 17, 28, 42, 47, 53, 55–57, 61, 62, 64, 66, 69].

**Fig. 1:**
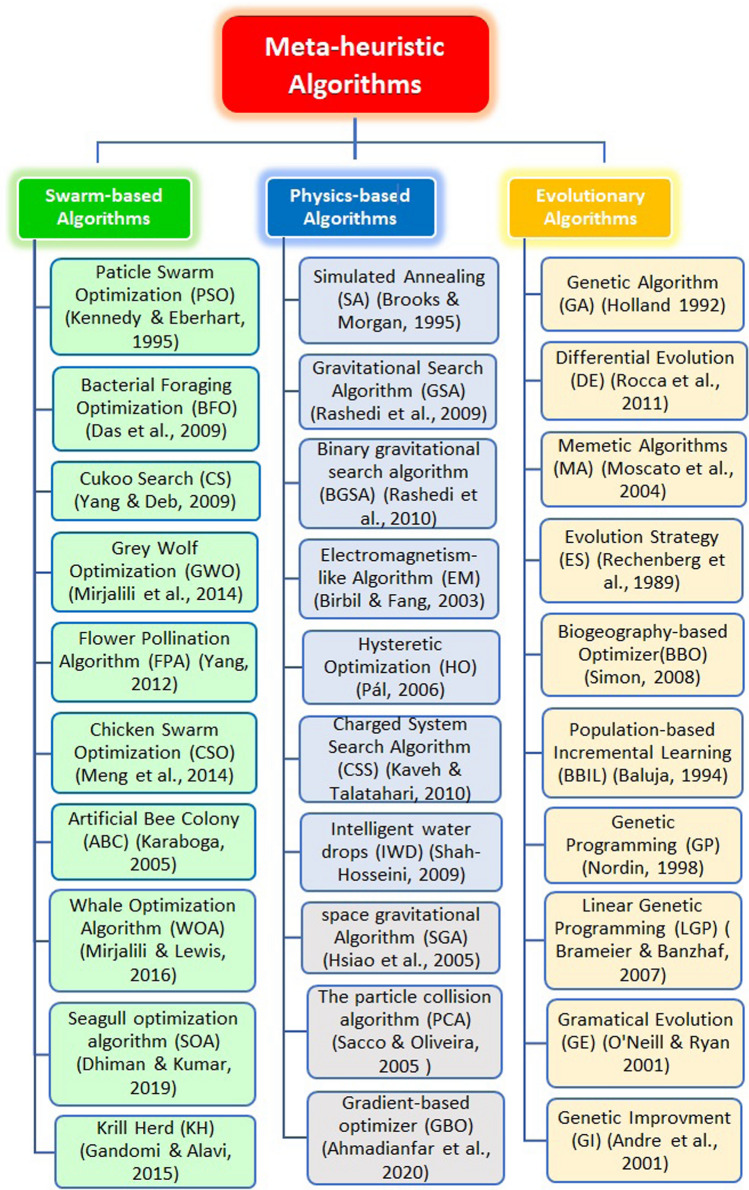
Most popular optimization algorithms

Generally speaking, optimization algorithms are classified into three categories: swarm-based, physics-based, and evolutionary algorithms. Swarm-based algorithms such as ABC, PSO, CSO, and CS, mimic how a group of agents would behave with each other and their environment [1]. Based on Newton's gravitational law, physics-based algorithms are based on a mathematical idea or physical processes, such as CFO and GSA [3]. On the other hand, evolutionary algorithms are search methods inspired by biological evolution mechanisms, such as reproduction and mutation [77]. The most popular evolutionary algorithm is GA, inspired by Darwin's theory of biological evolution. As mentioned in [23], evolutionary algorithms have some advantages over other types of optimization algorithms, such as:

1. They are conceptually simple: all evolutionary algorithms have similar necessary steps: initialization, fitness evaluation, selection, crossover, and mutation.
2. In evolutionary algorithms, the individuals with the highest fitness are selected for reproduction, leading to new individuals' production closer to the optimum solution.
3. Broad applicability: researchers can apply evolutionary algorithms to any problem formulated in the form of an optimization function. A list of the most popular nature-inspired algorithms is shown in Fig. 1.

Since 2020, the world has suffered from the pandemic of coronavirus disease 2019 (COVID-19). Researchers worldwide are doing their best to understand this novel virus's mechanism and find an effective therapy for this disease [40, 73]. More than one researcher discussed the mechanism of the novel Coronavirus from different perspectives in the optimization field. The authors in [47] proposed a bio-inspired metaheuristic algorithm based on the propagation model of coronavirus, and the experimental results showed quite remarkable performance of the algorithm. Al-Betal et al. [4] proposed an optimization algorithm based on herd immunity's effect in tackling the COVID pandemic. The comparative analysis showed that the proposed algorithm yields very competitive results compared to other well-established methods. Another algorithm [27] models the coronavirus distribution process as an optimization problem to minimize the number of COVID-19 infected countries and slow the epidemic.

Once the virus is inside the human body, the most severe problem is replication and transcription, in which new copies of the virus are created and target new healthy cells [49, 71]. This paper presents a novel evolutionary optimization algorithm named Coronavirus Disease Optimization Algorithm (COVIDOA).

COVIDOA mimics the attacking behavior of coronavirus inside human cells. It is worth mentioning that almost all kinds of viruses have the same general steps for replication: entry, uncoating, replication, assembly, and virion release. However, replication between viruses greatly varies depending on the genes involved [20].

In addition to the advantages of evolutionary algorithms, COVIDOA has several advantages when compared with other similar mechanisms:

1. Based on the virus's novelty and the lack of research on its various aspects, the reported numerical data about the coronavirus lifecycle may be inaccurate. Therefore, the proposed algorithm parameters, such as the number of virus particles in each generation and the number of viral proteins generated by each particle, haven't been set at fixed values. These reasons give the researchers’ flexibility to use the extendable values for the controlling parameters that most fit according to their problem.
2. As mentioned in [8], the mutation rate of coronavirus is 1 × 10^–6^, which is very low; however, the mutation rate in the proposed algorithm is set at a larger value in the range [0.1 0.001], which helps in exploring new promising regions and avoid getting stuck in a local minimum.
3. This study simulates a different virus replication technique known as the frameshifting technique [12, 43]. The virus uses frameshifting to create more copies of itself, leading to large-scale changes to polypeptide length and chemical composition. It is considered the most harmful to the molecular evolution of human cell proteins resulting in a non-functional protein that often disrupts the biochemical processes of a cell [59]. Applying the frameshifting technique in the proposed algorithm helps update solutions so that the solutions in each generation will not become too similar, which would allow the algorithm to converge to the global minimum.

The rest of the paper is structured as follows. Section 2 describes the inspiration and mathematical model of the proposed algorithm (COVIDOA). Experiments using test benchmark functions and the obtained results are discussed in Sect. 3. Finally, this study's conclusion and future work are presented in Sect. 4.

## Proposed algorithm

In this section, the inspiration and mathematical model of COVIDOA are presented.

### Inspiration

**Fig. 2:**
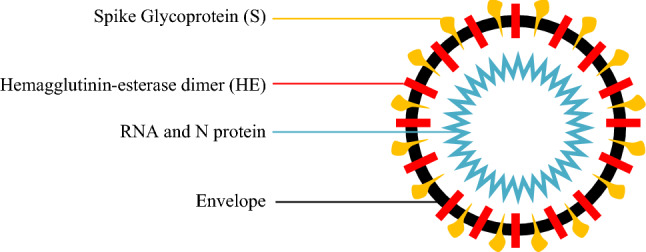
Structural proteins of COVID-19 (https://commons.wikimedia.org/wiki/File:3D_medical_animation_corona_virus.jpg) [30]

The new Coronavirus disease (COVID-19) is an infectious respiratory disease caused by Ssevere Aacute Respiratory Syndrome-CoronaVirus-2 (SARS-CoV-2). SARS-CoV-2 belongs to the coronaviruses family, named for crown-like spikes on their surface [34, 52], as shown in Fig. 2.

The coronavirus consists of a set of genetic instructions inside an oily membrane. These instructions are encoded in 30,000 letters of Ribonucleic Acid (RNA)—*a*, *c*, *g*, and *u*—then read by the infected cell and translated into many types of virus proteins [8]. Like other Coronaviruses, SARS-CoV-2 (COID-19) has four structural proteins, including the spike (*S*), envelope (*E*), and membrane (*M*) that constitute the viral coat, and the nucleocapsid (N) protein, which encapsulates the viral RNA [12]. Human-to-human transmission of SARS-CoV-2 occurs primarily via respiratory droplets from coughs and sneezes. Complications may include acute respiratory distress syndrome (ARDS), multi-organ failure, septic shock, and death [43].

The most serious problem of the virus is rapid replication, where it creates millions of copies of itself and sends it out to damage as many as possible human healthy cells. The replication mechanism of coronavirus inspires the proposed algorithm. For the virus to replicate, it passes through several stages as follows:

#### Virus entry and uncoating

**Fig. 3:**
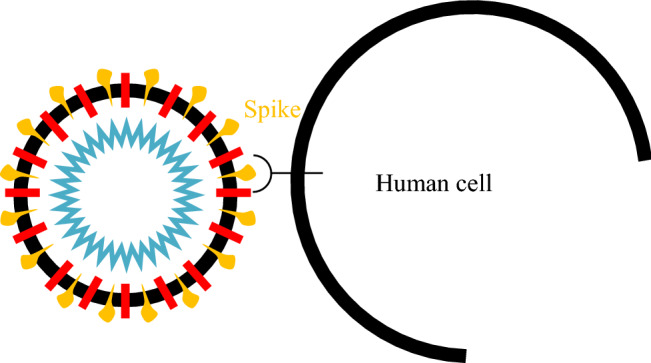
Virus attachment to human cell through spike protein (https://time.com/5839932/how-remdesivir-works-coronavirus/) [31]

**Fig. 4:**
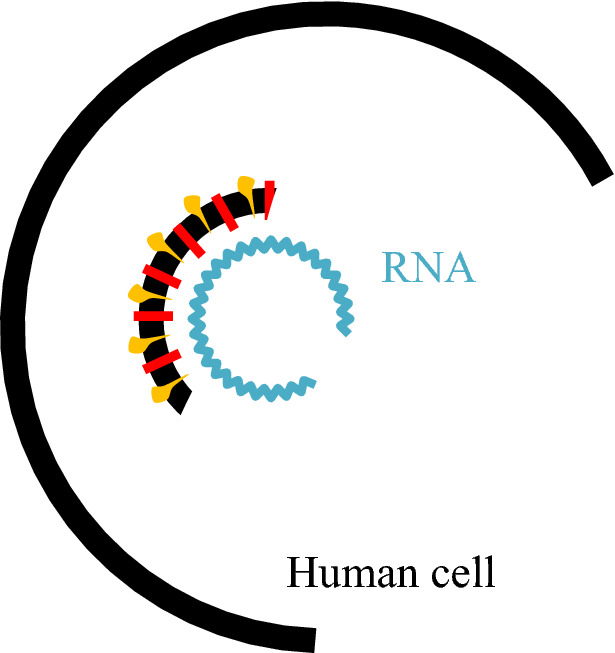
Virus entry and uncoating

For replication, coronavirus needs to use the human cell's protein-making machinery. So, it first needs to gain entry into the cell. The virus contains a set of spike (S) proteins; it uses its spike proteins as a key to getting inside a human cell [9, 72]. One spike of the virus binds to a protein called angiotensin-converting enzyme 2 (ACE-2) [67] on the surface of some human cells, as shown in Fig. 3. Coronavirus has a sort of membrane that hides its genetic material from the outside world; human cells have the same membrane that hides their material from the outside world. So, when those two things come together, the virus must find a way to get inside the host cell [65]. Once inside, all structural proteins are removed, and the virus contents, the genomic RNA, will be released into the host cell cytoplasm. This process is called virus uncoating [74], as shown in Fig. 4.

#### Virus replication

**Fig. 5:**
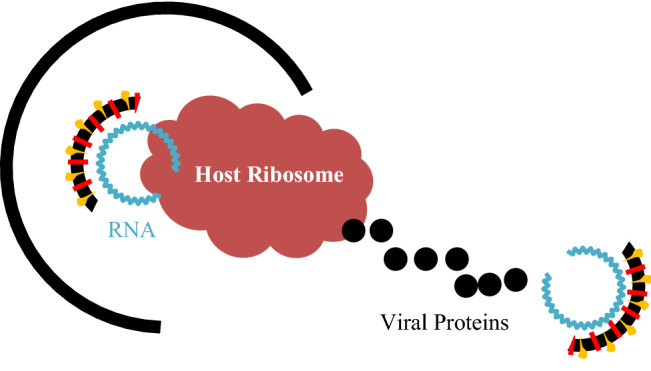
Virus RNA converts to viral proteins

Suppose the virus is getting fused in the cell membrane. Its small genetic material must hijack big cellular machinery in the next step. It will be tedious if the virus has few proteins to hijack the cell. The virus genome starts to find something in the host cell called a ribosome [79], a ribosome turns the virus RNA into many virus proteins through the ribosomal frameshifting technique [36], as shown in Fig. 5.

##### Ribosomal frameshifting during genome translation

Ribosomal frameshifting is also known as translational frameshifting, a biological phenomenon that occurs during translation [36, 54]. This phenomenon creates multiple unique proteins from a single messenger RNA (mRNA) molecule [14]. The translation is when the mRNA (messenger Ribonucleic Acid) molecule provides information to ribosomes, leading to protein molecules' formation [36, 37]. At the same time, frameshifting is when a specific reading frame of RNA molecule shifts to another reading frame to provide a new protein sequence [67, 72, 74]. To understand this, we need to understand translation and frameshifting separately.

**Fig. 6:**
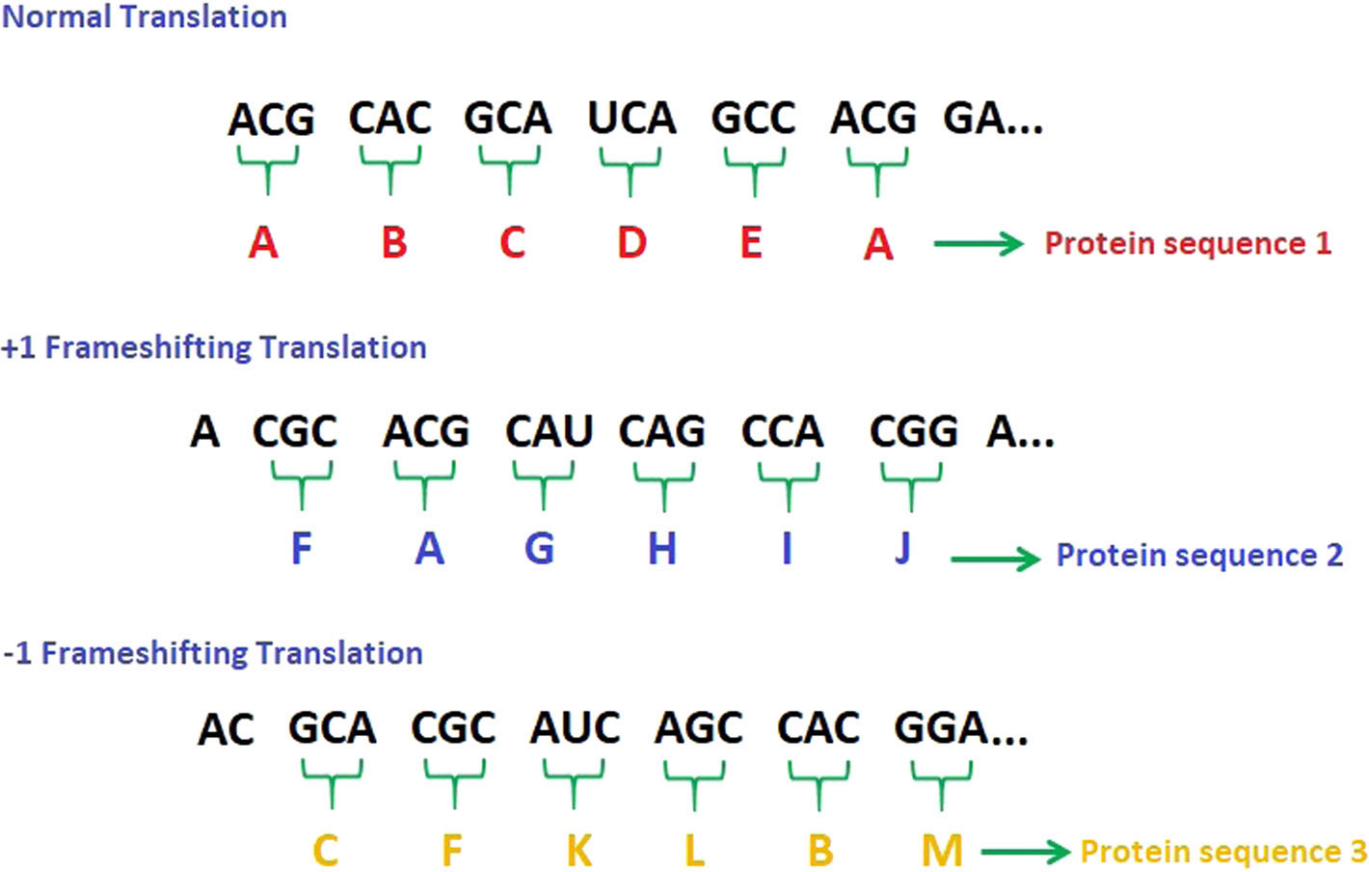
Generation of different protein sequences during frameshifting

The frameshifting technique is presented in Fig. 6. As shown in the figure, in the replication process, the virus's mRNA is translated into viral proteins by reading tri-nucleotides (e.g., ACG). Each tri-nucleotide is translated into single amino acid [52]. Thus, shifting (backward or forward) the reading frame of the nucleotides sequence by any number (not divisible by 3) will create different sequences that will be translated into different viral proteins [68].

Each group of the newly created viral proteins is merged to form a new virion. According to this technique, the virus can create millions of new particles than will damage millions of human cells.

In a translating ribosome, a frameshifting can result in either a nonsense mutation [68, 72] or a new protein after the frameshift. The most common types of frameshifting are − 1 frameshifting and + 1 frameshifting [58].

**Fig. 7:**
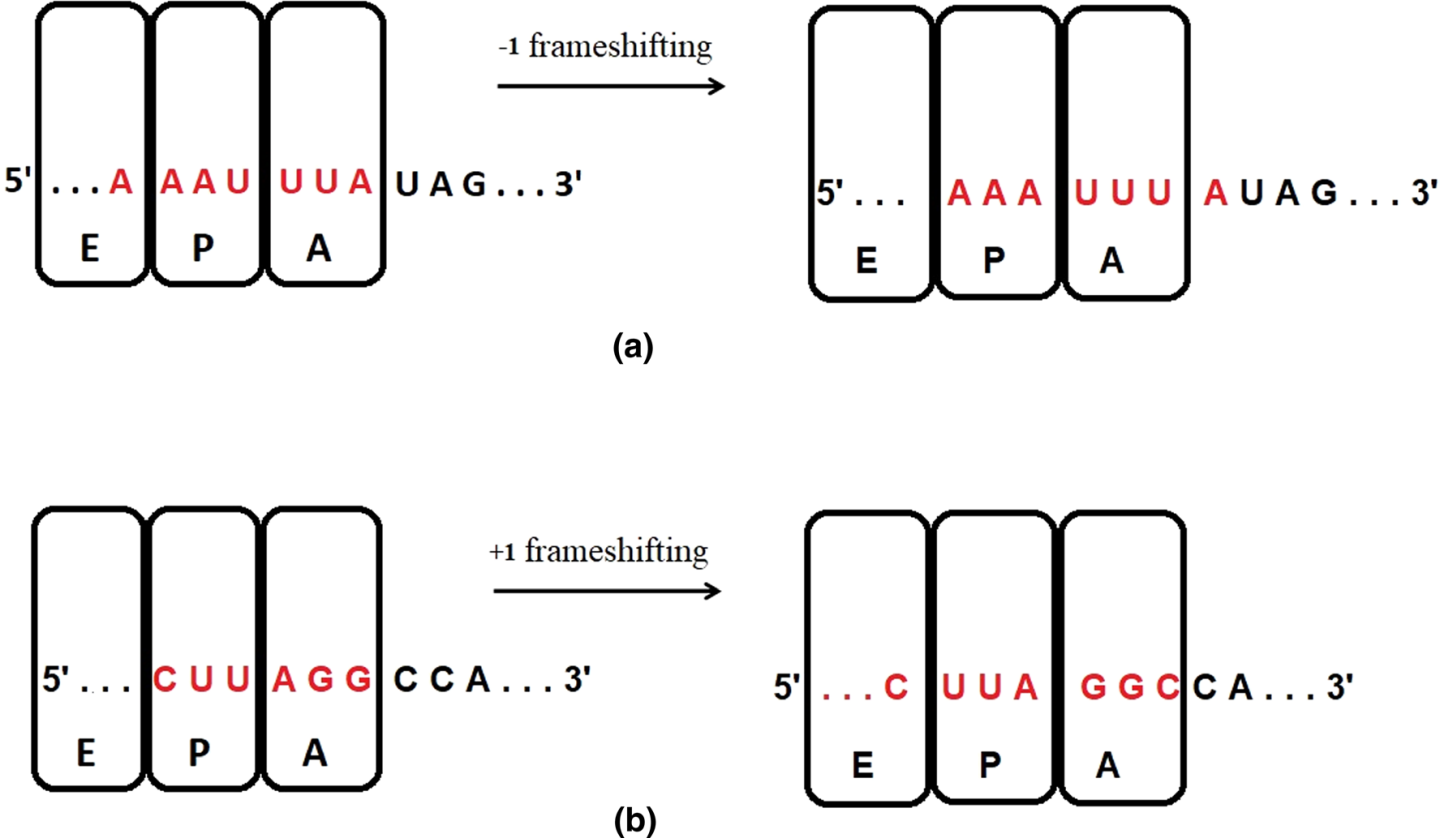
Different examples of frameshifting technique **a** − 1 frameshifting, **b** + 1 frameshifting, where E, P, and A, are the first, second, and third binding sites for RNA in the ribosome [14]

A. − 1 Frameshifting
  In − 1 frameshifting, the ribosome slips back one nucleotide (RNA letter) and continues translation in the − 1 frame, as shown in Fig. 7a.
B.  + 1 Frameshifting

The ribosome starts translation from the + 1 frame when 0 is the initial position, as shown in Fig. 7b. Because of shifting, the sequences are read differently and translated into different proteins.

##### Synthesis of both genomic and subgenomic RNA species

**Fig. 8:**
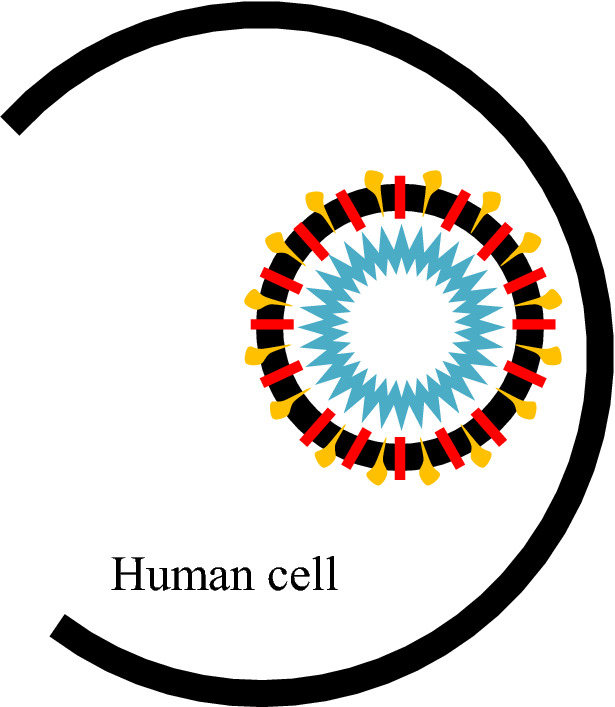
Release of the new virion. (https://time.com/5839932/how-remdesivir-works-coronavirus/)

The ribosomal frameshifting technique results in two types of RNAs, genomic RNA, and subgenomic RNAs. Genomic RNA is produced through the replication process and becomes the genome of the new virus particle. At the same time, Subgenomic RNAs are translated into many structural proteins (S: spike protein, E: envelope protein, M: membrane protein, and N: nucleocapsid protein). The genomic RNA and subgenomic RNAs are combined to form a viral particle [45, 58]. Finally, the new virion is released, trying to hijack new healthy cells, Fig. 8.

#### Virus mutation

**Fig. 9:**
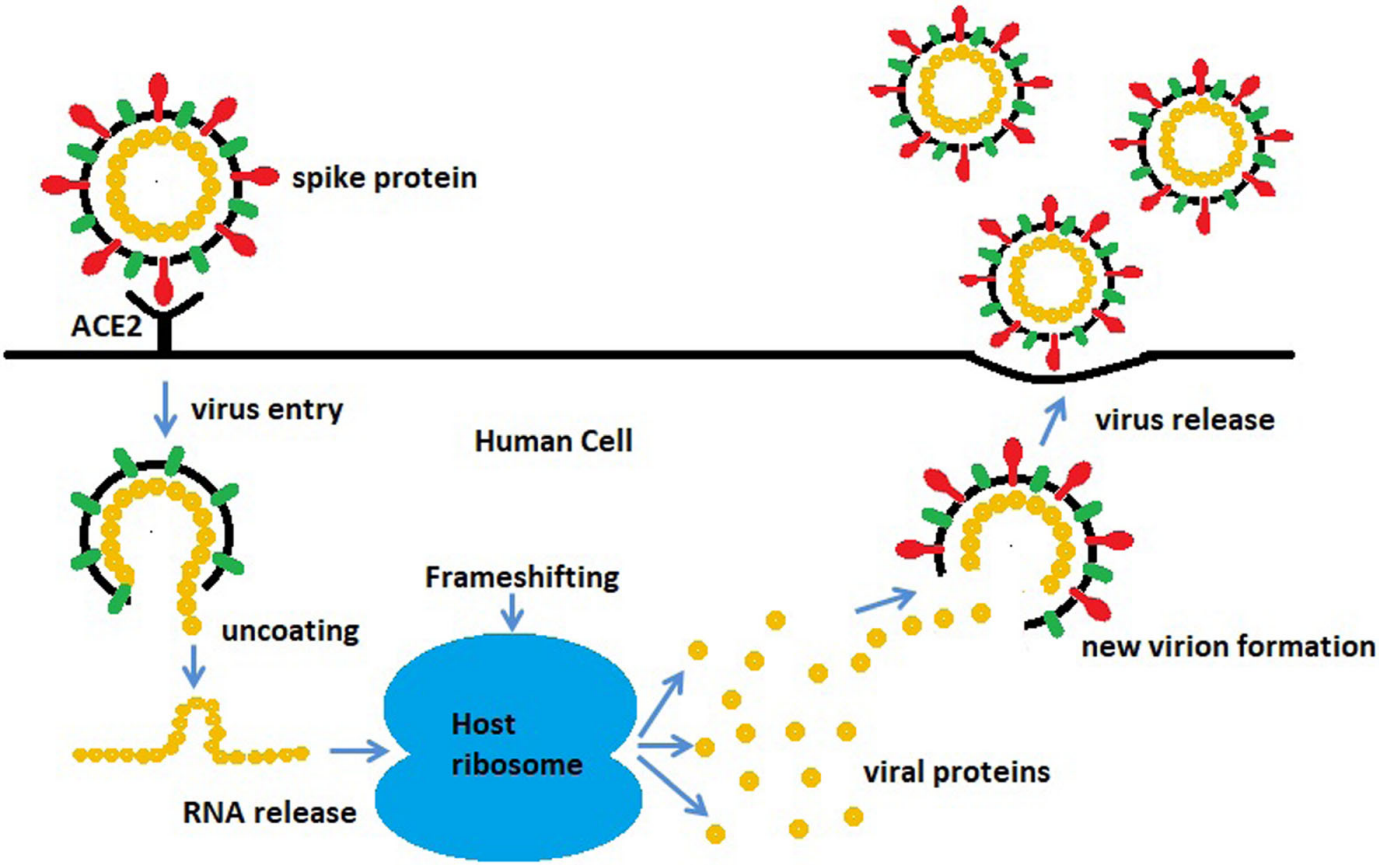
Replication lifecycle of coronavirus

As coronaviruses spread from person to person, they randomly accumulate more mutations to escape from the immune system [45]. Mutations involve changing one or more letters that represent the virus genome. As mentioned in [8], coronavirus has lower mutation rates (≈10^−6^ per site per cycle) in comparison with influenza (≈3 × 10^−5^ per site per cycle). The replication stages of coronavirus are summarized in Fig. 9.

### Mathematical model of COVIDOA

In this section, the mathematical model of COVIDOA is provided. COVIDOA is summarized in the following steps:

1. *Initialization* population of solutions is randomly initialized, and the cost is evaluated for each solution. The solutions are then ordered ascendingly according to the fitness function, and the first solution is considered the best solution.
2. *Virus replication phase through frameshifting technique* for each solution in the population, a parent is selected using roulette wheel selection [46] then,
  - The frameshifting technique is applied to produce several proteins from the selected parent as follows:
  - For each protein:
    i. If the + 1 frameshifting technique is used, the parent solution's values are shifted in the right direction by 1, and the value in the first position is set at a random value in the range [minVal maxVal] as follows.
      1 $$S_{k} \left( 1 \right) = {\text{rand}}\left( {{\text{minVal}},{\text{maxVal}}} \right),$$
      2 $$S_{k} \left( {2:D} \right) = P\left( {1:D - 1} \right),$$
      where minVal and maxVal are the minima and maximum values for the variables in each solution.
    ii. If the − 1 frameshifting technique is used, the parent solution values are shifted backward by 1, and the value in the last position is to set a random value in the range [minVal, maxVal].
      3 $$S_{k} \left( D \right) = {\text{rand}}\left( {{\text{minVal}},{\text{maxVal}}} \right),$$
      4 $$S_{k} \left( {1:D - 1} \right) = P\left( {2:D} \right),$$
      The symbol *S*_*k*_ refers to the *k*th generated protein, *P* is the parent solution, and *D* is the problem dimension (number of variables in each solution). The result of frameshifting represents a new protein sequence.
  - *New virion formation* a uniform crossover is applied to the generated sub-proteins to produce a new virion (new solution).
3. *Mutation* a mutation operator is applied to the solution created in the previous step to generate a new mutated solution as follows:
  5 $$Z\left( i \right) = \left\{ {\begin{array}{*{20}l} r \hfill & {{\text{if}}\;{\text{rand}}\left( {0,1} \right) < {\text{MR}}} \hfill \\ {X\left( i \right)} \hfill & {{\text{otherwise}}} \hfill \\ \end{array} } \right.$$
  *X* is the solution before mutation. *Z* is the mutated solution, $X\left( i \right)$ and $Z\left( i \right)$ are the *i*th element in the old and new solutions, respectively, *i* = 1, …, *D*, and *r* is a random value in the range [minVal, maxVal]. *MR* is the mutation rate.
4. The objective function is evaluated for the new solution, and the population is updated for the next generation (the solutions with the highest fitness remain, and the others are removed).
5. Repeat steps (2–4) for the new population until termination criteria are achieved. For example, the maximum number of iterations is reached.
6. Output the best solution found so far.

**Fig. 10:**
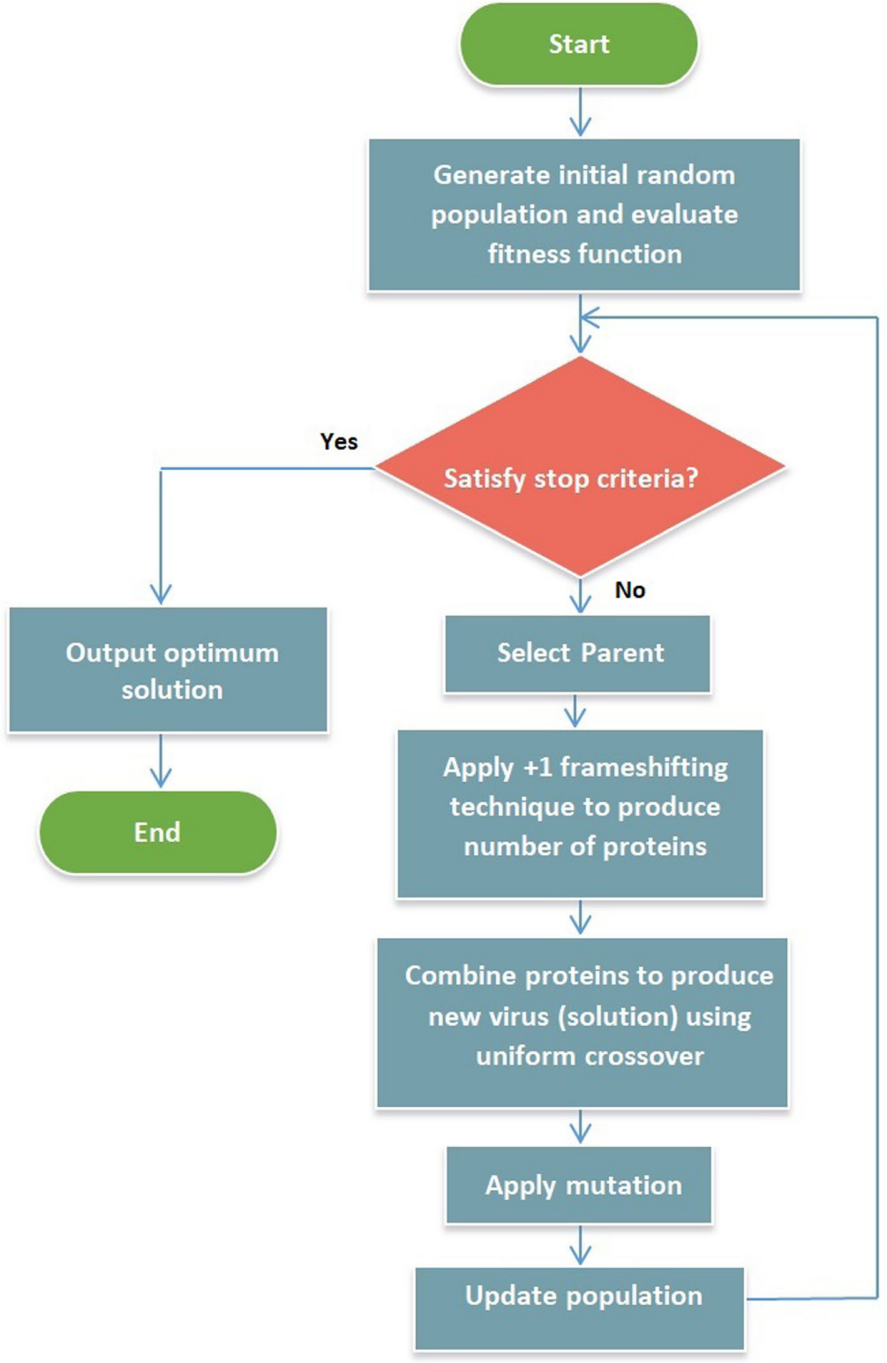
Flowchart of COVIDOA

The flowchart of the proposed algorithm is shown in Fig. 10.

### Parameters of the proposed algorithm

The parameters of the proposed algorithm are suggested as follows:

- *Max_Iter* maximum number of iterations.
- *PopNo* number of solutions in the population.
- *MinVal* and *MaxVal* minimum and maximum value of variables in a solution.
- *D* problem dimension (number of variables in each solution).
- *CostFunction* objective function;
- *MR* Mutation Rate, MR is set at a value in the range [0.1 0.001].
  The pseudocode of the proposed algorithm is as follows:

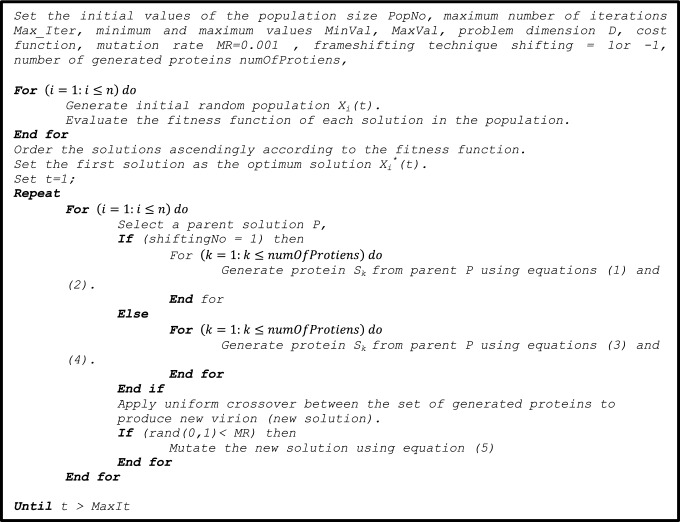

- *Shifting* a number that represents the type of frameshifting used. For example, shiftingNo = 1 means that the + 1 frameshifting technique is used. We noticed that the + 1 frameshifting technique yields the best results.
- *numOfProtiens* number of proteins generated during virus replication in the proposed algorithm, numOfProteins is 2.

## Experimental results and discussion

### Benchmark functions

To test the efficiency of the proposed COVIDOA, we utilized 30 benchmark functions. The first 20 are classical standard benchmark test functions (http://benchmarkfcns.xyz) [29]. We selected five functions from IEEE CEC 2019 Competition (https://www.mathworks.com/matlabcentral/fileexchange/72123-cec-06-2019-matlabimplementation) [33], while the remaining five were selected from CEC 2011 Competition on Testing Evolutionary Algorithms on Real-World Optimization Problems [16] as follows:

I. Classical benchmark problems

Twenty standard optimization functions from the literature are discussed and used to test the proposed algorithm's efficiency. These functions are classified into four groups: unimodal, multimodal, fixed-dimension, and n-dimensional functions [35, 39]. In fixed-dimension problems, the number of design variables (problem dimension) is fixed, while the other n-dimension problems use any design variables. A multimodal function has multiple (at least locally optimum) solutions instead of a unimodal function with a single optimum solution [35]. As in “Table 11 in the Appendix”, the chosen optimization functions are described in terms of the function name, formula, problem dimension (D), range of possible values, the global optimum, and the group of benchmark functions to which it belongs.

II. IEEE CEC 2019 benchmark problems

In addition to the classical benchmark functions, five CEC benchmark functions are utilized for evaluation. These are a group of modern test functions known as "The 100-Digit Challenge" intended to be used in single objective numerical optimization IEEE competitions [2]. As shown in “Table 12 in the Appendix”, these functions are described in terms of problem dimension, range of possible values, and the global optimum (https://www.mathworks.com/) [32].

III. CEC 2011 Real-World Problems

For further evaluation, COVIDOA was applied to five real-world optimization problems. These are bound-constrained real-world optimization problems selected from the CEC 2011 Competition on Testing Evolutionary Algorithms on Real-World Optimization. These problems are as follows [16]:

1. Lennard–Jones Potential Problem.
2. Transmission Network Expansion Planning (TNEP) problem.
3. Tersoff Potential Function Minimization Problem for model Si(B).
4. Tersoff Potential Function Minimization Problem for model Si(C).
5. Spread spectrum radar polyphase problem.

A detailed description of these real-world problems is discussed in the 2011 IEEE-Congress on Evolutionary Computation (IEEE-CEC 2011) [16].

### Experimental results

COVIDOA is utilized to solve the previously mentioned test problems. COVIDOA is implemented in MATLAB R2016a software. The results are compared with eight well-known and recent optimization algorithms: GA [26], DE [63], PSO [44], FPA [75], GWO [51], WOA [50], SOA [19], and CHIO [4]. We selected this group of algorithms for many reasons:

1. Most of them are recent and published in reputable sources.
2. All of them have high performance in single-objective optimization on various benchmark functions.
3. Their MATLAB implementations are publicly available on the MATLAB website (https://www.mathworks.com/) [32].
4. Some of them are evolutionary algorithms, such as GA and DE, in the same category as COVIDOA. CHIO algorithm simulates coronavirus, as is COVIDOA, but each has its inspiration.

The obtained results change at each run in optimization algorithms due to the random process. The commonly used number of runs is 30, which would give acceptable statistical precision. So, the proposed algorithm and the state-of-the-art algorithms are run 30 times.

The proposed and state-of-the-art algorithms use Max_Iter = 500 and PopNo = 1000 for the classical benchmark functions. The comparison is made regarding optimum cost, average cost, standard deviation (STD), and convergence speed. The authors downloaded the source code of the state-of-the-art optimization algorithms from the MATLAB website.

Tables 1, 2, 3 and 4 show the results of the best cost, average cost, standard deviation, and convergence speed, respectively, for the 20 classical benchmark functions. The best-obtained results in all the following tables are highlighted in bold. Table 1 shows that the proposed algorithm reaches the optimum global cost in 18 of 20 problems and gets very close to the global optimum in the two remaining problems. Table 2 proves the COVIDOA algorithm's efficiency in terms of the average cost. It reaches the minimum average cost in 17 from 20 problems and the second minimum average cost in three. The third criterion is STD, which shows how the cost values are far from the average cost. Low STD values mean the cost values over the iterations are clustered closely around the average cost. Table 3 shows that the COVIDOA algorithm reaches the minimum STD values in 17 of 20 problems, the second minimum in two, and the third minimum in two, which means that the results of COVIDOA are more reliable than the other algorithms with higher STD values.

**Table 1:** Best Cost results of COVIDOA and the state-of-the-art algorithms

Problem		Algorithm								
No.	Name	GA [26]	DE [63]	PSO [44]	FPA [75]	GWO [51]	WOA [50]	CHIO [4]	SOA [19]	Proposed COVIDOA
1	Dixon-price function	0.66667	0.40228	0.6667	4.9183	1	0.6667	1.694	0.6667	**0.27378**
2	Happy Cat function	0.1386	0.014702	0.24166	231.478	0.0122	1.4353	0.2691	0.005142	**0.0023146**
3	Crosslegtable function	− 0.08493	− 0.084778	− 0.07981	− 0.0006630	− 3.869e−04	− 0.0016362	− 2.606e−04	− 2.4310e−04	− **1**
4	Eggholder function	− 4886.18	− 7445.3819	− 5858.46	− 6292.2901	− 6.006 e+03	− 6319.4385	− 6385	− 5441.7	− **7825.143**
5	stybtang function	− 566.287	− **626.6587**	− 626.658	− 530.9072	− 626.086	− 555.9751	− 619.1	− 605.2622	− 626.621
6	Schwefel function	− 837.965	− 837.9529	− 837.965	− 837.9657	− 837.965	− **837.9658**	− 837.9548	− **837.9658**	− **837.9658**
7	Keane function	− **0.67367**	− **0.67367**	− **0.67367**	− **0.67367**	− 0.6736	− **0.67367**	− 0.6737	− **0.67367**	− **0.67367**
8	Trid function	− **2**	− **2**	− **2**	− **2**	− **2**	− **2**	− **2**	− **2**	− **2**
9	Schaffern4fcn function	0.2926	**0.29258**	**0.29258**	**0.29258**	0.2926	**0.29258**	0.2926	**0.29258**	**0.29258**
10	Branin function	**0.39789**	**0.39789**	**0.39789**	**0.39789**	**0.39789**	**0.39789**	0.4071	**0.39789**	**0.39789**
11	Wolfe function	**0**	**0**	**0**	**0**	**0**	**0**	**0**	**0**	**0**
12	Zettl function	− 0.0037	− 0.0037	− 0.0037	− 0.0037	− **0.0038**	− **0.0038**	− 0.0037	− **0.0038**	− **0.0038**
13	Alpine N. 2 function	− 14,320.0	− **23,700.87**	− 14,320.08	− 8649.361	− 2369	− 23,700.7978	− 1.7386	− 14,277	− 23,563.73
14	Cross-in-Tray function	− **2.0626**	− **2.0626**	− **2.0626**	− **2.0626**	− **2.0626**	− **2.0626**	− **2.0626**	− **2.0626**	− **2.0626**
15	McCormick function	− **1.9105**	− **1.9105**	− **1.9105**	− **1.9105**	− **1.9105**	− **1.9105**	− **1.9105**	− **1.9105**	− **1.9105**
16	Gramacy and Lee function	− **2.8739**	− **2.8739**	− **2.8739**	− **2.8739**	− **2.8739**	− **2.8739**	− **2.8739**	− **2.8739**	− **2.8739**
17	Testtubeholder function	− **10.8723**	− **10.8723**	− **10.8723**	− **10.8723**	− **10.8723**	− **10.8723**	− **10.8723**	− **10.8723**	− **10.8723**
18	Shubert function	− **186.7309**	− **186.7309**	− **186.7309**	− **186.7309**	− **186.7309**	− **186.7309**	− 186.7082	− **186.7309**	− **186.7309**
19	price 2 function	**0.9**	**0.9**	**0.9**	0.9004	**0.9**	**0.9**	0.9001	**0.9**	**0.9**
20	Dejong5	**0.998**	**0.998**	**0.998**	**0.998**	**0.998**	**0.998**	**0.9980**	**0.998**	**0.998**

**Table 2:** Average Cost results of COVIDOA and the state-of-the-art algorithms

Problem		Algorithm								
No.	Name	GA [26]	DE [63]	PSO [44]	FPA [75]	GWO [51]	WOA [50]	CHIO [4]	SOA [19]	Proposed COVIDOA
1	Dixon-price function	15.3545	126.5770	6.3509	1.0998e+03	46.6686	30.0319	1.0734e+03	9.897e+03	**5.23636**
2	Happy Cat function	0.6517	0.0445	0.2636	371.4819	0.0802	20.4486	0.2930	0.0477	**0.0137**
3	Crosslegtable function	− 0.0683	− 0.0427	− 0.0427	− 0.7909	− 5.1528e−04	− 2.6865e−04	− 2.182e−04	− 0.0047	− **0.8980**
4	Eggholder function	− 4.70e+03	− 6.75e+03	− 5.628e+03	− 5.681e+03	− 5.2816e+03	− 6.2799e+03	− 5.679e+03	− 4.262e+03	− **7.23e**+**03**
5	Stybtang function	− 393.6128	− 619.9509	− 619.2246	− 475.8865	− 577.2454	− 552.6846	− 572.8967	− 594.1131	− **622.7337**
6	Schwefel function	− 835.3788	− 821.9348	− 837.8732	− 837.5112	− 837.5351	− 837.9275	− 835.5825	− 837.6662	− **837.9367**
7	Keane function	− 0.673659	− **0.673667**	− **0.673667**	− 0.67359	− 0.673661	− 0.673633	− 0.6736	− 0.673519	− **0.673667**
8	Trid function	− 1.9999	− 1.9999	− 1.9999	− **2**	− 1.9999	− 1.9999	− 1.9996	− 1.9993	− **2**
9	Schaffern4fcn function	0.2928	0.2930	**0.2926**	0.2930	0.2927	0.2928	0.2961	0.2947	**0.2926**
10	Branin function	0.3980	0.3982	**0.3979**	0.3984	0.3987	0.3984	0.4673	0.4205	0.3981
11	Wolfe function	0.0144	1.7214e−04	8.5733e−05	**0**	3.3785e−04	1.4367e−04	0.0055	3.7236e−04	**0**
12	Zettl function	− **0.0038**	− **0.0038**	− **0.0038**	− 0.0036	− **0.0038**	− **0.0038**	− 0.0028	− 0.0036	− **0.0038**
13	Alpine N. 2 function	− 1.32e+04	− 2.114e+04	− 1.402e+04	− 5.826e+03	− 1.2565e+0	− 2.1515e+04	− 9.569e+03	− 2.014e+03	− **2.18e**+**04**
14	Cross-in-Tray function	− **2.0626**	− **2.0626**	− **2.0626**	− **2.0626**	− **2.0626**	− **2.0626**	− **2.0626**	− **2.0626**	− **2.0626**
15	McCormick function	− **1.9105**	− **1.9105**	− **1.9105**	− **1.9105**	− **1.9105**	− **1.9105**	− **1.9103**	− **1.9105**	− **1.9105**
16	Gramacy and Lee function	− **2.87389**	− 2.87384	− **2.87389**	− 2.**87389**	− **2.87389**	− **2.87389**	− 2.8739	− 2.87385	− **2.87389**
17	Testtubeholder function	− 10.8718	− 10.8720	− **10.8721**	− 10.8718	− **10.8721**	− 10.8717	− 10.8697	− 10.8638	− **10.8721**
18	Shubert function	− 186.6132	− 186.6495	− 186.6853	− 186.4929	− 186.6285	− 186.6954	− 186.4249	− 186.2621	− **186.7009**
19	Price 2 function	0.90037	0.900945	**0.900233**	0.902144	0.9006	0.90033	0.9031	0.91701	0.90004
20	Dejong5	1.0115	1.0065	0.9987	1.0218	1.0122	1.0100	1.1783	1.2333	**0.9980**

**Table 3:** STD results of COVIDOA and the state-of-the-art algorithms

Problem		Algorithm								
No.	Name	GA [26]	DE [63]	PSO [44]	FPA [75]	GWO [51]	WOA [50]	CHIO [4]	SOA [19]	Proposed COVIDOA
1	Dixon-price function	269.8620	1.1364e+03	62.4047	3.545e+03	894.3655	451.6185	4.0605e+03	7.220e+03	**52.7791**
2	Happy Cat function	0.1139	78.6462	46.1026	109.0887	0.0522	0.0406	0.0390	0.2955	**0.0294**
3	crosslegtable function	0.0321	0.0392	0.0357	1.557e−04	4.8352e−05	2.212e−04	3.1194e−05	**2.6201**e−**05**	2.8268e−05
4	Eggholder function	380.0907	878.5967	426.2801	574.4192	450.2399	**236.1962**	701.8892	979.6934	425.3867
5	Stybtang function	36.0487	42.5232	**13.4428**	45.6925	26	21.2045	49.1493	50.9105	18.6986
6	Schwef function	3.5750	0.3603	0.2791	1.9761	0.6225	2.0103	3.2773	6.3983	**0.1706**
7	Keane function	9.276e−05	4.1333e−06	3.492e−06	0.0011	1.242e−04	7.286e−04	7.1552e−05	0.0033	**6.6663**e−**08**
8	Trid function	0.0015	9.1279e−04	8.566e−05	3.721e−04	5.869e−04	0.0023	7.1631e−04	0.0023	**1.9900**e−**05**
9	Schaffern4fcn function	9.484e−04	0.0033	0.0469	0.0016	6.954e−04	0.0031	0.0050	0.0041	**5.6588**e−**04**
10	Branin function	0.0016	0.0013	3.902–04	0.0035	0.0063	0.0023	0.4256	0.0246	**3.6041**e−**04**
11	Wolfe function	0.0393	0.0027	0.0019	**0**	0.0076	0.0032	0.0303	0.0083	**0**
12	Zettl function	1.696e−04	2.3912e−04	6.959e−05	0.0011	8.467e−04	1.704e−04	0.0034	0.0015	**1.1646**e−**04**
13	Alpine N. 2 function	5.807e+03	2.1124e+04	**1.247e**+**03**	2.4308e+03	6.94e+034	3.901e+03	5.3061e+03	2.3812e+03	1.7739e+03
14	Cross-in-Tray function	3.718e−05	2.8873e−05	4.8930e−06	3.0880e−04	3.903e−05	3.9988e−05	1.4093e−04	0.0012	**4.5473**e−**06**
15	McCormick function	6.450e−05	1.3749e−04	1.361e−06	8.3451e−05	0.0013	7.382e−04	0.0013	0.0041	**2.6101**e−**07**
16	Gramacy and Lee function	4.198e−04	0.0010	1.856e−05	7.1457e−06	4.162e−04	6.207e−05	4.8450e−05	4.3901e−04	**4.1554**e−**08**
17	Testtubeholder function	0.0034	0.0018	**0.0015**	0.0065	0.0038	0.0058	0.0103	0.0207	0.0021
18	shubert function	0.6984	0.5832	0.4346	1.2720	1.2720	0.3873	0.7193	0.7625	**0.2339**
19	Price 2 function	0.0034	0.0068	0.0045	0.0063	0.0054	0.0050	0.0096	0.0375	**1.4613**e−**04**
20	Dejong5	0.1339	0.1025	8.7297e−05	0.1555	0.1185	0.1910	0.4304	0.7947	**3.4653**e−**05**

**Table 4:** Convergence speed of COVIDOA and the state-of-the-art algorithms

Problem		Algorithms								
No.	Name	GA [26]	DE [63]	PSO [44]	FPA [75]	GWO [51]	WOA [50]	CHIO [4]	SOA [19]	Proposed COVIDOA
1	Dixon-price function	**Moderate**	**Moderate**	**Moderate**	Slow	**Moderate**	**Moderate**	**Moderate**	Slow	**Moderate**
2	Happy Cat function	**Moderate**	Slow	**Moderate**	Slow	Slow	Slow	Slow	Slow	**Moderate**
3	Crosslegtable function	Moderate	Moderate	Moderate	Slow	Slow	Slow	Slow	Slow	**Fast**
4	Eggholder function	Slow	Slow	Slow	Slow	Slow	Slow	Slow	Slow	**Moderate**
5	Stybtang function	Slow	Slow	Slow	Slow	Slow	Fast	**Fast**	Slow	**Fast**
6	Schwef function	**Fast**	**Fast**	**Fast**	**Fast**	**Fast**	**Fast**	Moderate	**Fast**	**Fast**
7	Keane function	**Fast**	**Fast**	**Fast**	**Fast**	**Fast**	**Fast**	**Fast**	**Fast**	**Fast**
8	trid function	**Fast**	**Fast**	**Fast**	**Fast**	**Fast**	**Fast**	**Fast**	Slow	**Fast**
9	schaffern4fcnfunction	**Fast**	**Fast**	**Fast**	**Fast**	**Fast**	**Fast**	**Fast**	Moderate	**Fast**
13	Alpine N. 2 function	Slow	**Moderate**	Slow	Slow	**Moderate**	Slow	**Moderate**	Slow	**Moderate**
14	Cross-in-Tray function	**Fast**	**Fast**	**Fast**	**Fast**	**Fast**	**Fast**	**Fast**	Slow	**Fast**
15	McCormick function	**Fast**	**Fast**	**Fast**	**Fast**	**Fast**	**Fast**	**Fast**	**Fast**	**Fast**
18	Shubert function	**Fast**	**Fast**	**Fast**	**Fast**	**Fast**	**Fast**	**Fast**	**Fast**	**Fast**
19	Price 2 function	**Fast**	**Fast**	**Fast**	Moderate	**Fast**	**Fast**	**Fast**	**Fast**	**Fast**
20	Dejong5	**Fast**	**Fast**	**Fast**	**Fast**	**Fast**	**Fast**	**Fast**	Moderate	**Fast**

**Fig. 11:**
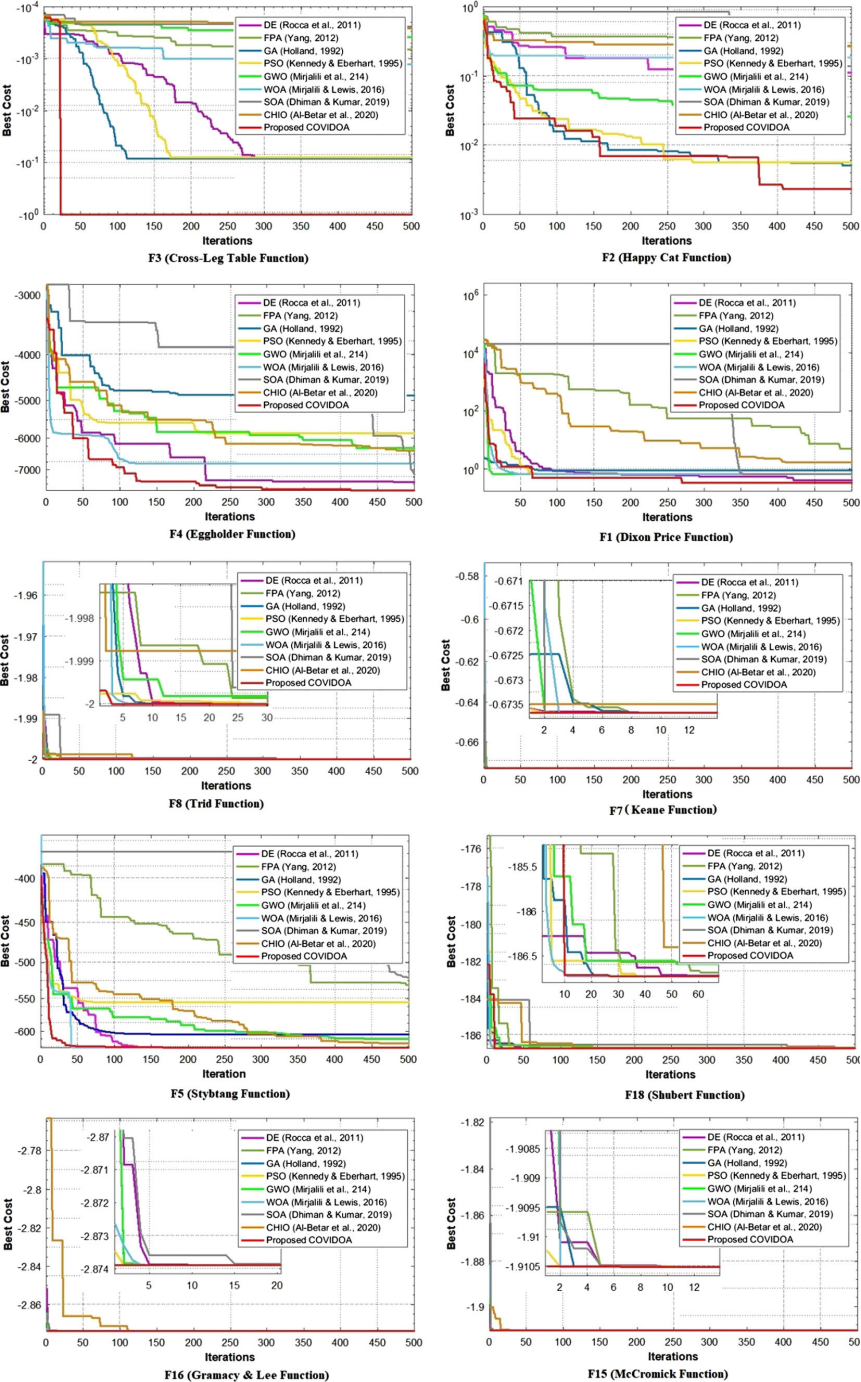
Comparison of convergence curves of COVIDOA and state-of-the-art algorithms for group 1 of the test problems

**Fig. 12:**
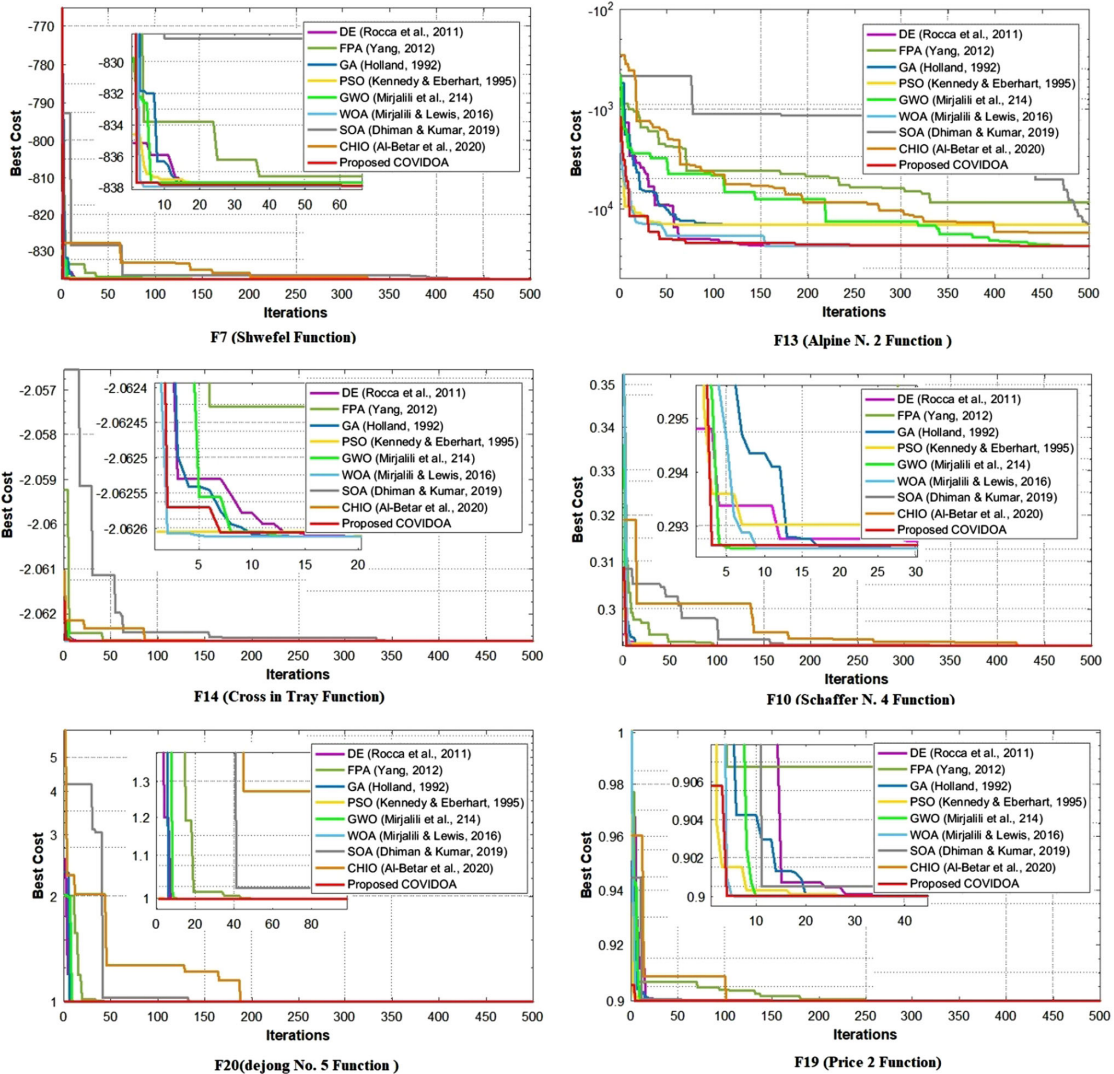
Comparison of convergence curves of COVIDOA and state-of-the-art algorithms for group 2 of the test problems

**Fig. 13:**
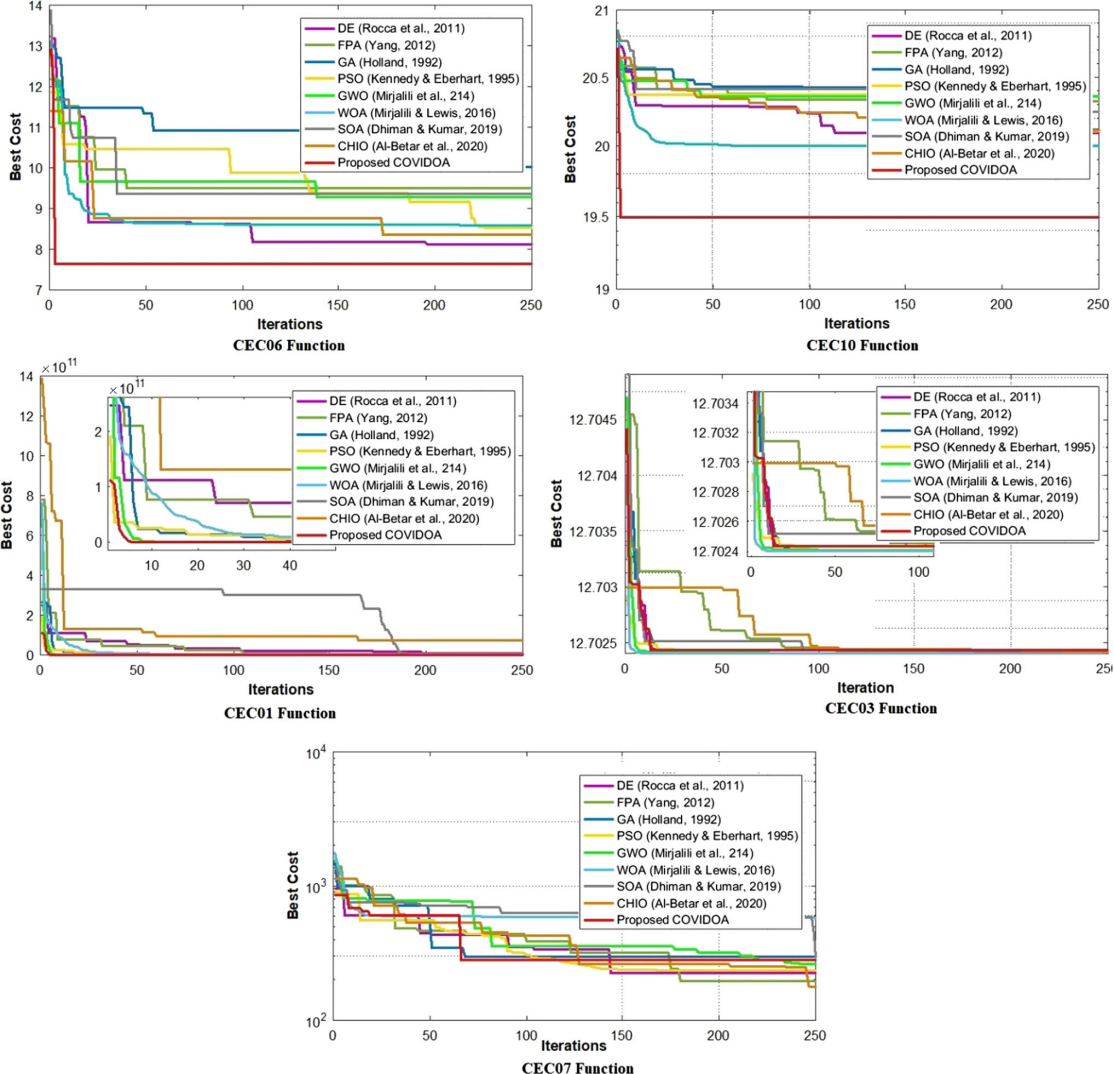
Comparison of convergence curves of COVIDOA and state-of-the-art algorithms for CEC benchmark functions

**Fig. 14:**
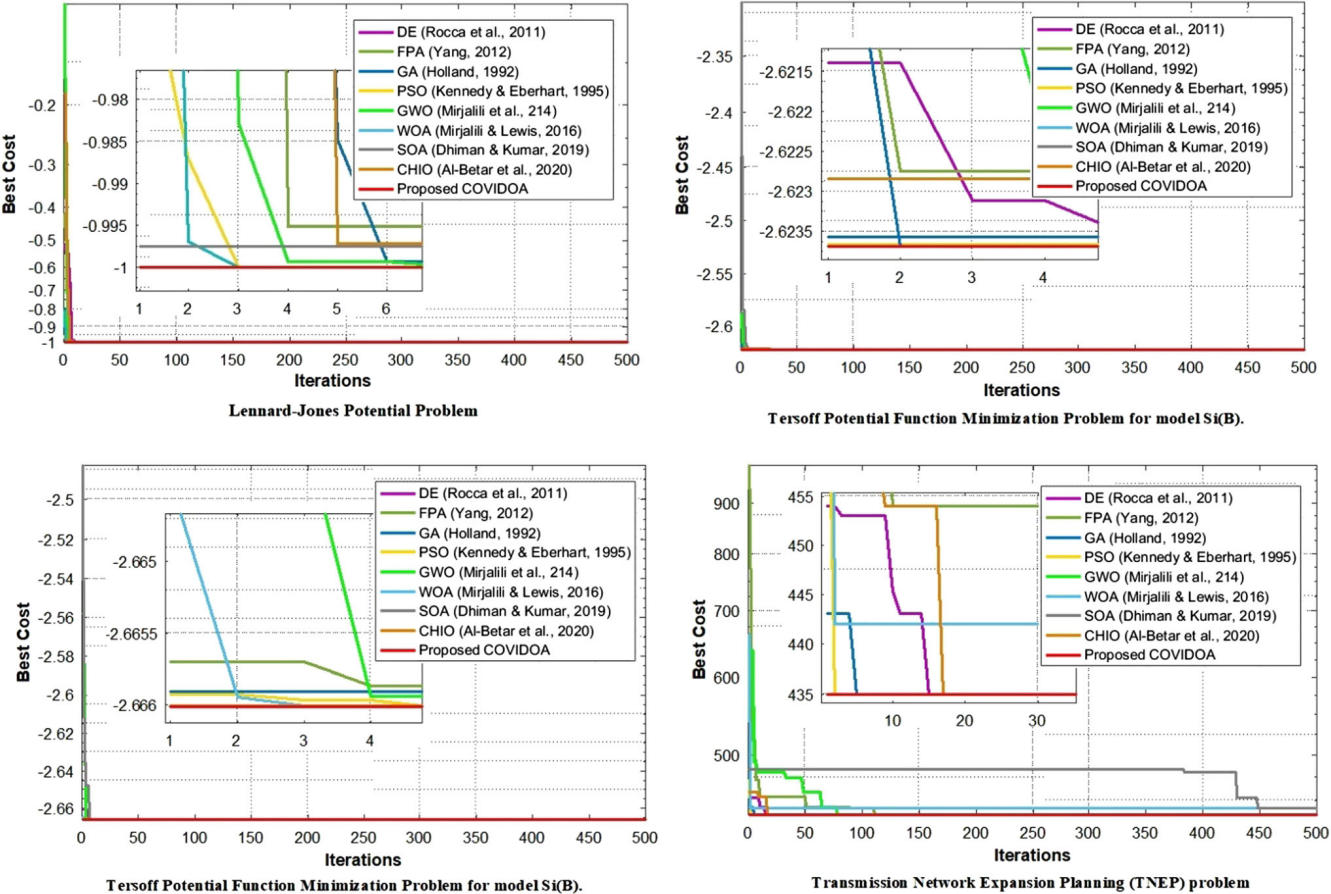
Comparison of convergence curves of COVIDOA and state-of-the-art algorithms for CEC 2011 real-world problems

Compared with the recently proposed algorithm, CHIO, which simulates herd immunity's effect in tackling the COVID pandemic, COVIDOA is the best. As shown in Tables 1, 2, 3 and 4 and Figs. 11, 12, 13 and 14, CHIO reaches the minimum optimum cost in seven benchmark functions only from 25; in contrast, COVIDOA reaches the minimum optimum cost in 21 from 25 test functions. This indicates that COVIDOA has robust exploration capabilities in comparison with CHIO.

Compared with PSO, GWO, and WOA, COVIDOA is superior according to most of the test problems' best cost, average cost, and STD values. It has a higher convergence speed as it reaches the global minimum after the first few iterations, as in functions (F3, F8, F7, F15, and F16).

The curves in Figs. 11 and 12 represent the relationship between the iterations and the corresponding best cost for the classical test functions. The obtained results using the selected test problems are divided into two groups and displayed in Figs. 11 and 12. Figure 11 represents the test problems for which the COVIDOA algorithm outperforms the other algorithms. In contrast, Fig. 12 shows the results of test problems in which the COVIDOA algorithm has a performance very close to the others.

Additionally, to prove the results' statistical significance, the test results of the 20 classical benchmark functions are compared using Wilcoxon rank-sum test at the 5% significance level [18]. A null hypothesis is a type of hypothesis used in statistics that assumes no significant difference between the two methods' average values. A small *p*-value (typically ≤ 0.05) indicates strong evidence against the null hypothesis [70].

Table 5 introduces the *p* values computed by Wilcoxon rank-sum test that compares the COIDOA with eight well-known metaheuristic algorithms for the 20 classical benchmark functions. We observed from Table 5 that all *p* values are less than a 5% significance level for all comparative algorithms, strong evidence against the null hypothesis. Therefore, we conclude that the COVIDOA is better than all other comparative algorithms.

**Table 5:** P values computed by Wilcoxon's rank-sum test compared the COVIDOA with other algorithms for 20 classical benchmark functions

Problem		Algorithm							
No.	Name	COVIDOA vs. GA	COVIDOA vs. DE	COVIDOA vs. PSO	COVIDOA vs. FPA	COVIDOA vs. GWO	COVIDOA vs. WOA	COVIDOA vs. CHIO	COVIDOA vs. SOA
1	Dixon-price function	2.2242e−06	8.0835e−24	1.3497e−09	1.6207e−129	6.6181e−12	1.0616e−13	4.0517e−134	**2.2667**e−**102**
2	Happy Cat function	2.3444e−41	2.9609e−142	4.6130e−59	7.0570e−151	7.8828e−78	6.2314e−153	3.3083e−158	**2.3994**e−**168**
3	Crosslegtable function	3.8478e−147	2.6216e−149	1.2983e−131	5.9989e−71	8.8060e−112	3.0525e−168	1.7277e−168	**2.1944**e−**5**
4	Eggholder function	1.5930e−08	3.5431e−35	4.7752e−101	6.9415e−107	4.2077e−102	1.2085e−97	7.0318e−99	**1.8854**e−**169**
5	stybtang function	3.5988e−89	3.8579e−92	9.8348e−156	3.5551e−160	1.3910e−150	4.2362e−151	4.8158e−148	**7.4638**e−**172**
6	Schwefel function	1.3895e−123	1.9569e−164	4.7482e−132	2.6478e−24	2.7706e−42	1.3129e−155	1.9594e−121	**7.4398**e−**142**
7	Keane function	2.1931e−145	4.5728e−141	7.8435e−146	7.4084e−141	1.8957e−138	2.5242e−139	4.3658e−155	**1.3654**e−**145**
8	Trid function	2.3005e−04	1.2665e−07	5.4793e−15	3.9880e−12	3.6942e−132	2.7804e−129	2.0510e−140	**7.0287**e−**143**
9	Schaffern4fcn function	8.6497e−151	1.4164e−133	1.2696e−139	3.4423e−57	8.8837e−158	1.5795e−160	5.5992e−53	**1.0205**e−**04**
10	Branin function	1.4628e−170	1.5300e−166	9.9148e−147	3.9973e−56	4.0798e−31	1.5096e−163	3.7413e−04	**1.4814**e−**99**
11	Wolfe function	8.6069e−11	1.6745e−18	1.3438e−25	1.3438e−25	8.1128e−25	8.2198e−25	3.2408e−05	**8.2198**e−**25**
12	Zettl function	2.4618e−47	3.7395e−48	7.9714e−46	4.2188e−51	8.4116e−43	2.6398e−43	6.3415e−64	**2.3241**e−**60**
13	Alpine N. 2 function	1.1328e−63	2.1170e−87	4.9124e−160	9.4075e−169	2.3117e−98	5.4748e−96	5.4748e−96	**3.0303**e−**170**
14	Cross-in-Tray function	2.9415e−190	6.3621e−190	9.4571e−165	2.9144e−118	1.6057e−167	4.4898e−185	8.7420e−20	**7.1009**e−**30**
15	McCormick function	4.8145e−208	7.9734e−199	2.4157e−205	2.7981e−185	1.7211e−193	3.8569e−208	1.5483e−54	**1.6457**e−**188**
16	Gramacy and Lee function	5.0302e−214	1.1779e−213	1.7334e−200	2.6517e−189	2.1106e−212	1.5681e−192	2.3659e−191	**3.2889**e−**191**
17	Testtubeholder function	1.3355e−161	2.0663e−138	1.4054e−120	6.2333e−26	2.0588e−151	1.2910e−163	1.4419e−48	**9.7121**e−**15**
18	Shubert function	7.8405e−182	4.1448e−96	2.8226e−121	3.6690e−18	5.0688e−103	1.7861e−161	4.6324e−105	**1.9701**e−**164**
19	price 2 function	5.8287e−19	2.4336e−06	1.7689e−07	2.1156e−119	1.6710e−31	1.1040e−24	3.8123e−70	**3.1442**e−**18**
20	Dejong5	1.7349e−183	6.1675e−188	2.6969e−179	7.4328e−178	3.8529e−175	5.8155e−177	4.8973e−178	**3.7030**e−**182**

CEC benchmark functions, COVIDOA, and state-of-the-art algorithms search for the optimum cost for 250 iterations with 1000 solutions in each generation. The results of the best cost, average cost, and STD values are discussed in Table 6, and the convergence curves are shown in Fig. 13. COVIDOA is superior to the other algorithms in CEC01, CEC06, and CEC01. The CEC03 problem reaches the minimum best cost and the second minimum average cost ad STD value. In the case of CEC07, however, it is not the best; it achieves excellent results compared to GA, FPA, GWO, WOA, SOA, and CHIO algorithms.

**Table 6:** Best, average, and STD results of COVIDOA and the state-of-the-art algorithms for CEC benchmark functions

Problem	Metric	Algorithm								
		GA [26]	DE [63]	PSO [44]	FPA [75]	GWO [51]	WOA [50]	CHIO [4]	SOA [19]	Proposed COVIDOA
CEC01	Best	4.79e+07	8.067e+09	2.130e+08	2.525e+09	6.58e+06	4.585e+09	7.011e+06	7.35e+10	**1.25e**+**06**
	AVG	7.767e+09	3.648e+10	4.108e+09	3.4008e+10	4.260e+09	1.623e+10	2.2465e+11	1.294e+11	**1.044e**+**09**
	STD	3.649e+10	3.729e+10	1.394e+10	8.531e+10	4.8333e+10	5.6522e+10	1.3991e+11	1.755e+11	**6.249e**+**09**
CEC03	Best	**12.7024**	**12.7024**	**12.7024**	**12.7024**	**12.7024**	**12.7024**	**12.7024**	**12.7024**	**12.7024**
	AVG	12.**7024**	12.7025	**12.7024**	12.7026	**12.7024**	**12.7024**	12.7025	12.7028	12.7025
	STD	1.8779e−04	2.3779e−04	**4.8999**e−**05**	3.7993e−04	1.8226e−04	1.0041e−04	2.5001e−04	5.063e−04	9.8359e−05
CEC06	Best	10.0164	7.7598	8.5145	9.4978	9.2790	7.7528	9.3672	8.0529	**7.6402**
	AVG	10.7198	8.7656	9.7656	9.7070	9.5928	8.6969	9.6018	9.2519	**8.6512**
	STD	0.6542	8.6156	0.7421	0.5951	0.5048	1.2646	0.6372	0.8336	**0.4291**
ECE07	Best	296.0888	**165.6218**	242.9147	176.8028	305.1	546.7268	277.5	317.7	276.0837
	AVG	409.9065	**265.9382**	388.3867	334.7019	461.5165	570.3746	316.4750	566.4644	376.4779
	STD	231.6249	186.9450	266.6071	159.2303	168.3036	176.3487	**119.4227**	170.8287	163.8042
CEC10	Best	20.1179	20.0925	20.1074	20.3277	20.3589	20.0006	20.2471	20.1112	**19.4927**
	AVG	20.4208	20.1859	20.2848	20.3669	20.3789	20.0226	20.3975	20.2414	**19.4976**
	STD	0.0823	0.1245	0.1128	0.0686	0.0697	0.0863	0.0933	0.1412	**0.0574**

All test results for the CEC benchmark functions were compared using the Wilcoxon rank-sum test to prove their statistical significance. Table 7 shows the *p* values computed by Wilcoxon rank-sum test that compares the COIDOA with other well-known algorithms for CEC benchmark functions. It is evident from Table 7 that all *p* values are less than 5% which proves the statistical significance of COVIDOA.

**Table 7:** P values computed by Wilcoxon's rank-sum test compared the COVIDOA with other algorithms for CEC benchmark functions

Problem	Algorithm							
	COVIDOA vs. GA	COVIDOA vs. DE	COVIDOA vs. PSO	COVIDOA vs. FPA	COVIDOA vs. GWO	COVIDOA vs. WOA	COVIDOA vs. CHIO	COVIDOA vs. SOA
CEC01	2.0762e−19	3.9935e−44	4.0173e−28	1.2177e−28	7.4696e−24	3.5076e−25	9.3679e−33	9.7806e−73
CEC03	4.3959e−10	2.0317e−06	2.8802e−14	6.8629e−04	1.6530e−18	2.6432e−19	2.8370e−17	1.8324e−08
CEC06	9.1167e−05	7.2701e−19	1.7786e−05	4.3378e−31	7.0423e−26	2.0190e−23	1.8914e−35	2.6879e−18
ECE07	2.8384e−16	3.5116e−19	5.2696e−12	3.7006e−13	2.8596e−09	6.4990e−32	6.5814e−21	6.1956e−26
CEC10	6.4014e−13	1.8025e−19	1.0889e−28	5.0020e−28	3.9464e−26	8.7411e−19	4.3551e−32	1.4533e−20

To test the impact of changing parameter values on the performance of OVIDOA, we used nine different scenarios by changing the values of the parameters MR (Mutation Rate) and numOfProtiens. We utilized the values of 0.1, 0.01, ad 0.001 for MR, 2, 4, and 6 for numOfProtiens which produces nine scenarios, as shown in Table 8. The results of each scenario on the selected five IEEE CEC benchmark problems are presented in Table 9. We noticed that scenario 1 (MR = 0.1 and numOfProtiens = 2) has better results, followed by scenario 4. The common between these two scenarios is MR = 0.1 which represents a higher mutation rate. This comparison shows that higher MR values are better for improving the performance of the proposed algorithm.

**Table 8:** Scenarios of the tuning parameters

Scenario	Parameters	
	MR	numOfProtiens
1	0.1	2
2	0.01	2
3	0.001	2
4	0.1	4
5	0.01	4
6	0.001	4
7	0.1	6
8	0.01	6
9	0.001	6

**Table 9:** The impact of COVIDOA parameters (MR, a numOfProtiens) on IEEE CEC problems

Problem	Metric	Scenario 1	Scenario 2	Scenario 3	Scenario 4	Scenario 5	Scenario 6	Scenario 7	Scenario 8	Scenario 9
CEC01	Best	8.41 e+07	1.93e+08	4.84 e+08	**7.22e**+**06**	8.94e+08	4.36e+08	6.64e+08	6.31e+08	6.10e+08
	AVG	6.90e+09	7.34e+09	5.21e+09	**6.15e**+**08**	4.82e+09	4.47e+09	1.03e+10	9.49e+09	6.44e+09
	STD	1.75e+10	2.38e+10	1.51e+10	1.99e+10	1.80e+10	**1.20e**+**10**	3.22e+10	4.06e+10	1.79e+10
CEC03	Best	**12.7024**	**12.7024**	**12.7024**	12.7025	12.7025	12.7025	12.7025	12.7025	12.7025
	AVG	**12.7025**	**12.7025**	**12.7025**	**12.7025**	12.7026	12.7026	**12.7025**	12.7026	12.7026
	STD	2.08e−04	**8.34**e−**05**	1.26e−04	2.91e−04	3.03e−04	2.17e−04	1.60e−04	1.14e−04	2.29e−04
CEC06	Best	**7.6402**	9.0038	9.3928	9.0148	8.344	7.7169	8.8126	8.7189	9.1483
	AVG	**8.6512**	9.7509	9.9594	9.4252	8.9131	9.2187	8.8800	9.2262	9.6000
	STD	**0.4291**	1.0948	0.9469	0.5378	0.8895	1.2194	0.4741	0.7438	0.5159
ECE07	Best	429.593	467.8152	525.5403	**276.0837**	388.5537	508.3128	455.5922	560.6439	404.9701
	AVG	**478.6812**	600.8125	699.5776	493.5525	460.4821	640.0468	468.0766	719.8709	521.5527
	STD	135.4222	139.3537	101.7200	202.0033	137.9164	157.4621	**87.5660**	145.9606	218.8682
CEC10	Best	**19.3901**	20.241	20.3035	20.301	20.104	20.2851	20.3317	20.2618	20.2906
	AVG	**20.1491**	20.3671	20.3797	20.3672	20.2833	20.3194	20.3450	20.3261	20.3269
	STD	0.2631	0.0939	0.0591	**0.0478**	0.1631	0.0615	0.0531	0.1012	0.0788

For testing COVIDOA on CEC real-world problems, we obtain our results over 500 iterations. The proposed and state-of-the-art algorithms were run 25 independent times as suggested by IEEE-CEC 2011 Competition [16]. Table 10 and Fig. 14 show the results of the selected CEC real-world problems. The proposed algorithm achieves the optimum best cost, average cost, and STD values for all five selected problems.

**Table 10:** The best, average, and STD results of COVIDOA and the state-of-the-art algorithms for CEC 2011 real-world problems

Problem	Meric	Algorithms								
		GA [26]	DE [63]	PSO [44]	FPA [75]	GWO [51]	WOA [50]	CHIO [4]	SOA [19]	COVIDOA
Lennard–Jones Potential Problem	Best	− **1**	− **1**	− **1**	− **1**	− **1**	− **1**	− **1**	− **1**	− **1**
	AVG	− 0.9984	− 0.9949	− 0.9999	− 0.9968	− 0.9967	− 0.9996	− 0.9998	− 0.9953	− **1**
	STD	0.0177	0.0461	0.0019	0.0409	0.0516	0.0087	5.7843e−04	0.0549	**4.8460**e−**07**
Spread spectrum radar polyphase problem	Best	**0.5**	**0.5**	**0.5**	**0.5**	**0.5**	**0.5**	**0.5**	**0.5**	**0.5**
	AVG	0.5004	**0.5**	**0.5**	0.5014	0.5003	0.5007	**0.5**	0.5002	**0.5**
	STD	0.0056	**0**	**0**	0.0096	0.0040	0.0094	**0**	0.0018	**0**
Tersoff Potential function Minimization Problem for model Si(B)	Best	− **2.6237**	− **2.6237**	− **2.6237**	− **2.6237**	− **2.6237**	− **2.6237**	− **2.6237**	− **2.6237**	− **2.6237**
	AVG	− 2.6237	− 2.6237	− 2.6237	− 2.6235	− 2.6236	− 2.6237	− **2.6228**	− 2.6236	− 2.6237
	STD	1.3471e−05	1.5074e−04	3.3914e−05	0.0036	0.0016	2.5855e−04	0.0145	2.3704e−04	**2.7394**e−**07**
Tersoff Potential function Minimization Problem for model Si(C)	Best	− **2.666**	− **2.666**	− **2.666**	− **2.666**	− **2.6660**	− **2.6660**	− **2.6660**	− **2.666**	− **2.666**
	AVG	− 2.6660	− 2.6660	− 2.6660	− 2.6660	− 2.6656	− 2.6660	− **2.6654**	− 2.6660	− 2.6660
	STD	1.8503e−05	5.0644es− 04	6.2625e−06	2.8149e−05	0.0068	7.0142e−05	0.0088	2.0518e−06	**5.8070**e−**07**
Transmission Network Expansion Planning (TNEP) problem	Best	**435**	**435**	**435**	**435**	**435**	442	442	442	**435**
	AVG	435.0640	435.4125	435.0740	440.3519	441.4249	442.4413	478.2145	435.6794	**435**
	STD	0.7134	2.5585	1.6547	32.7886	21.9935	9.8685	13.7593	3.7615	**0**

Although the general steps of COVIDOA and other evolutionary algorithms, such as GA and DE, are very similar, COVIDOA is superior to them, as shown in Tables 1, 2, 3, 4, 5, 6, 7, 8, 9 and 10. This progress is caused by the additional step proposed in the replication phase of COVIDOA, the frameshifting technique. Adding frameshifting technique in the replication process helps COVIDOA update solutions in each generation, helping to reach global optimum rapidly.

### Explorations and exploitation capabilities of COVIDOA

It is essential to test the efficiency of the proposed algorithm. In other words, it is necessary to test its exploration and exploitation capabilities. In exploration, the algorithm searches for new solutions in new regions, while exploitation means using existing solutions and improving their fitness [21]. Mutation and crossover steps in COVIDOA are used to create new solutions, so they are methods to explore the problem space. On the other hand, selecting an existing parent virus and applying the frameshifting technique to produce new children represents the exploitation of the current solution features. Unimodal test functions F1, F7, F10, and F13 can evaluate the exploitation feature because they have only one global optimum solution. Multimodal functions F2, F3, F4, and F5 can help assess the exploration capability of COVIDOA because they have many optimum solutions.

### Convergence of COVIDOA

In Table 4, the convergence speed of COVIDOA and the other algorithms for the classical benchmark functions are classified into three groups: Fast, Moderate, and Low, where algorithms that reach the minimum cost in the first 100 iterations are classified as fast convergence algorithms, those that get the minimum cost from iteration 100 to 300 are moderate convergence algorithms, and the others classified as slow convergence algorithms. As shown in Table 5 and Fig. 13, the proposed algorithm has fast convergence in the majority (16 from 20) of the test problems and moderate in the others. In contrast, other state-of-the-art algorithms may slow the test problems' convergence.

Overall results reveal that COVIDOA reaches the minimum best cost, average cost, and standard deviation in most test problems. It also has high exploration and exploitation capabilities and a high convergence speed during iterations.

## Conclusion

A novel evolutionary optimization algorithm (COVIDOA) inspired by the replication lifecycle of SARS-CoV-2 is presented. The proposed COVIDOA was tested by solving 20 classical benchmark problems, five CEC benchmark test functions, and five CEC 2011 real-world problems. The proposed COVIDOA is compared with the state-of-the-art nature-inspired optimization algorithms in terms of best cost, average cost, standard deviation, and convergence speed. The proposed algorithm is implemented using MATLAB R2016a software, and the source code of the state-of-the-art algorithms and the benchmark problems are downloaded from the mathworks.com website. The experimental results proved that the proposed algorithm outperforms the state-of-the-art optimization algorithms in most test problems and has very close results to other algorithms in the rest of the test problems. COVIDOA has high exploitation and exploration capabilities and convergence speed compared to other metaheuristics.

Future work may include the implementation of COVIDOA in solving large-scale problems in different fields.

## Data Availability

The datasets generated during and/or analyzed during the current study are available from the corresponding author on reasonable request.

## Appendix

See Tables

**Table 11:** Description of classical benchmark functions

Function	Formula	Dimension (D)	Range	Global optimum cost	Properties
Dixon− price function	$F_{1} \left( x \right) = \left( {x_{1} - 1} \right)^{2} + \mathop \sum \limits_{i = 2}^{d} i\left( {2x_{i}^{2} - x_{i - 1} } \right)^{2}$	D	*X*_*i* _∈ [− 10, 10], for all *i* = 1, …, *d*	0	Unimodal nD function
Happy Cat function	$$\begin{aligned} F_{2} \left( x \right) & = \left[ {\left( {x^{2} - n} \right)^{2} } \right]^{\alpha } \\ & \quad + \frac{1}{n}\left( {\frac{1}{2}x^{2} + \mathop \sum \limits_{i = 1}^{n} x_{i} } \right) + \frac{1}{2} \\ & {\text{Where}}\;\alpha = \frac{1}{8} \\ \end{aligned}$$	D	*X*_*i*_ ∈ [− 2,2], for all *i* = 1, …, *d*	0	Multimodal nD function
Cross-Leg Table function	$F_{3} \left( {\text{x}} \right) = \frac{1}{{\left( {\left| {{\text{sin}}\left( {x_{1} } \right){\text{sin}}\left( {x_{2} } \right)} \right|e^{{\left| {100 - \frac{{\sqrt {x_{1}^{2} + x_{2}^{2} } }}{\pi }} \right|}} + 1} \right)^{0.1} }}$	2	*X*_*i* _∈ [− 10, 10], *i* = 1,2	− 1	Multimodal 2D function
Eggholder function	$F_{4} \left( x \right) = - \left( {x_{2} + 47} \right)\sin \left( {\sqrt {\left| {x_{2} + \frac{{x_{1} }}{2} + 47} \right|} } \right) - x_{1} sin\left( {\sqrt {\left| {x_{1} - x_{2} + 47} \right|} } \right)$	2	*X*_*i* _∈ [− 5.12, 5.12], *i* = 1, 2	− 959.6407	Multimodal 2D function
Alpine N. 2 function	$F_{5} \left( x \right)\mathop \prod \limits_{i = 1}^{n} \sqrt {x_{i} } \sin \left( {x_{i} } \right)$	D	*X*_*i* _∈ [0, 10], for all *i* = 1,* d*	2.808^d^	Multimodal nD function
Styblinski-tang function	$F_{6} \left( {\text{x}} \right) = \frac{1}{2}\mathop \sum \limits_{i = 1}^{d} \left( {x_{i}^{4} - 16x_{i}^{2} + 5x_{i} } \right)$	D	*X*_*i* _∈ [− 5, 5], for all *i* = 1, …, *d*	− 39,16599d	Multimodal nD function
Schwefel function	$F_{7} \left( x \right) = 418.9829 - \mathop \sum \limits_{i = 1}^{d} x_{i} \sin \left( {\sqrt {\left| {x_{i} } \right|} } \right)$	D	*x*_*i*_ ∈ [− 500, 500], for all *i* = 1, …, *d*	0	Unimodal nD function
Keane function	$F_{8} \left( x \right) = \frac{{\sin^{2} \left( {x_{1} - x_{2} } \right)\sin^{2} \left( {x_{1} + x_{2} } \right)}}{{\sqrt {x_{1}^{2} + x_{2}^{2} } }}$	2	*x*_*i* _∈ [0, 10], *i* = 1,2	0.6736675	Multimodal 2D function
Trid function	$F_{9} \left( x \right) = \mathop \sum \limits_{i = 1}^{d} \left( {x_{i} - 1} \right)^{2} - \mathop \sum \limits_{i = 2}^{d} x_{i} x_{i - 1}$	D	*x*_*i*_ ∈ [− *d*^2^, *d*^2^], for all *i* = 1, …, *d*	$\frac{{ - d\left( {d + 4} \right)\left( {d - 1} \right)}}{6}$	Multimodal nD function
Schaffer function n. 4	$F_{10} \left( x \right) = 0.5 + \frac{{\cos^{2} \left( {\sin \left( {\left| {x_{1}^{2} - x_{2}^{2} } \right|} \right)} \right) - 0.5}}{{\left( {1 + 0.001\left( {x_{1}^{2} + x_{2}^{2} } \right)} \right)^{2} }}$	2	*x*_*i*_ ∈ [− 100, 100], *i* = 1,2	0.292579	Unimodal 2D function
Branin function	$$\begin{aligned} F_{11} \left( x \right) & = a\left( {x_{2} - bx_{1}^{2} + cx_{1} - r} \right)^{2} \\ & \quad + {\text{s}}\left( {1 - {\text{t}}} \right)\cos \left( {{\text{x}}_{1} } \right) + {\text{s}} \\ \end{aligned}$$ The recommended values of a, b, c, r, s and t are: a = 1, b = 5.1/(4π2), c = 5/π, r = 6, s = 10 and t = 1/(8π)	2	*x*_1_ ∈ [− 5, 10], *x*_2_ ∈ [0, 15]	0.397887	Multimodal 2D function
Wolfe function	$F_{12} \left( {x,y,z} \right) = \frac{4}{3}\left( {x^{2} + y^{2} - xy} \right)^{0.75} + z$	3	*x*_*i*_ ∈ [− 65.536, 65.536], *i* = 1, 2	0.998	Multimodal 2D function
Zettl function	$F_{13} \left( x \right) = \left( {x_{0}^{2} { } + { }x_{1}^{2} - { }2x_{0} } \right)^{2} { } + { }0.25x_{0}$	2	*x*_*i*_ ∈ [− 5, 5], *i* = 1, 2	− 0.003791	Unimodal 2D function
Cross-in-Tray function	$F_{14} \left( x \right) = 0.0001\left( {\left| {\sin \left( {x_{1} } \right)\sin \left( {x_{2} } \right)\exp \left( {\left| {100 - \frac{{\sqrt {x_{1}^{2} + x_{2}^{2} } }}{\pi }} \right|} \right)} \right| + 1} \right)^{0.1}$	2	*x*_*i*_ ∈ [− 10, 10], *i* = 1, 2	− 2.06261	Multimodal 2D function
McCormick function	$$\begin{aligned} F_{15} \left( x \right) & = \sin \left( {x + y} \right) + \left( {x - y} \right)^{2} \\ & \quad - 1.5x + 2.5y + 1 \\ \end{aligned}$$	2	*x*_1_ ∈ [− 1.5,4], And *x*_2_ ∈ [− 3,3]	− 1.9133	Multimodal 2D function
Gramacy and Lee function	$F_{16} \left( x \right) = \sin \frac{{10{\pi x}}}{2x} + \left( {x + 1} \right)^{4}$	1	*x* ∈ [− 0.5,2.5]	− 0.8690111349	Multimodal 1D function
Test tube holder function	$$F_{17} \left( x \right) = - 4\left[ {\left( {\sin \left( {x1} \right)\cos \left( {x2} \right)e^{{\left| {\cos \left[ {\left( {{\raise0.7ex\hbox{${x_{1}^{2} + x_{2}^{2} }$} \!\mathord{\left/ {\vphantom {{x_{1}^{2} + x_{2}^{2} } {200}}}\right.\kern-\nulldelimiterspace} \!\lower0.7ex\hbox{${200}$}}} \right)} \right]} \right|}} } \right)} \right]$$	2	*x*_*i*_ ∈ [− 10, 10], *i* = 1, 2	− 10.872300	Multimodal 2D function
Shubert function	$$\begin{aligned} F_{18} \left( x \right) & = \left( {\mathop \sum \limits_{i = 1}^{5} \alpha_{i} i \cos \left( {\left( {i + 1} \right)x_{1} + i} \right)} \right) \\ & \left( {\mathop \sum \limits_{i = 1}^{5} \alpha_{i} i \cos \left( {\left( {i + 1} \right)x_{2} + i} \right)} \right) \\ \end{aligned}$$	2	*x*_*i*_ ∈ [− 10, 10], *i* = 1, 2	− 186.7309	Multimodal nD function
Price 2 function	$F_{19} \left( x \right) = 1 + \sin^{2} \left( {x_{1} } \right) + \sin^{2} \left( {x_{2} } \right) - 0.1e^{{ - x_{1}^{2} - x_{2}^{2} }}$	2	*x*_*i*_ ∈ [− 10, 10], *i* = 1, 2	0.9	Multimodal 2D function
De Jong function n. 5	$F_{20} \left( x \right) = \left( {0.002\mathop \sum \limits_{i = 1}^{25} \frac{1}{{i + \left( {x_{1} + a_{1i} } \right)^{6} + \left( {x_{2} + a_{2i} } \right)^{6} }} } \right)^{ - 1} ,$ where $$a = \left( {\begin{array}{*{20}c} { - 32} & { - 16} \\ { - 32} & { - 32} \\ \end{array} \begin{array}{*{20}c} 0 & {16} \\ { - 32} & { - 32} \\ \end{array} \begin{array}{*{20}c} {32} & { - 32} \\ { - 32} & { - 16} \\ \end{array} \ldots \begin{array}{*{20}c} 0 & {16} & {32} \\ {32} & {32} & {32} \\ \end{array} } \right)$$	2	*x*_*i*_ ∈ [− 65.536 65.536], *i* = 1, 2	0	Multimodal 2D function

11 and

**Table 12:** Description of CEC benchmark functions

No.	Function	Dimension	range	Global minimum
CEC01	Storn’s chebychev polynomial fitting problem	9	[− 8192, 8192]	1
CEC03	Lennard–Jones minimum energy cluster	18	[− 4, 4]	1
CEC06	Weierstrass function	10	[− 100, 100]	1
CEC07	Modified Shwefel function	10	[− 100, 100]	1
CEC10	Ackley function	10	[− 100, 100]	1

^12^.
